# Intestinal parasites in stool testing among refugees at a primary care clinic in Toronto, Canada

**DOI:** 10.1186/s12879-022-07226-4

**Published:** 2022-03-13

**Authors:** Frank Müller, Shivani Chandra, Isaac I. Bogoch, Meb Rashid, Vanessa Redditt

**Affiliations:** 1 Crossroads Clinic, Women’s College Hospital, 76 Grenville St., Toronto, ON M5S 1B2 Canada; 2 Department of General Practice, University Medical Center Göttingen/Georg-August-University, Humboldtallee 38, 37073 Göttingen, Germany; 3 Institute for Health System Solutions and Virtual Care (WIHV), Women’s College Hospital, 76 Grenville Street, Toronto, ON M5S 1B2 Canada; 4 Department of Medicine, University of Toronto, Toronto, ON Canada; 5 Divisions of General Internal Medicine and Infectious Diseases, Toronto General Hospital, 14EN 209, 200 Elizabeth Street, Toronto, ON M5G 2C4 Canada; 6 Department of Family and Community Medicine, University of Toronto, 500 University St, Toronto, ON M5G 1V7 Canada

**Keywords:** Parasites, Protozoans, Helminths, Refugees, Stool, Worm

## Abstract

**Background:**

Enteric parasites are endemic in many of the countries from which refugees originate. Clinical guidelines vary in approaches to screening for and treating intestinal parasites in refugee receiving countries. This study aims to investigate the prevalence and species of intestinal parasites identified in stool ova and parasite (O&P) specimens in a sample of newly arrived refugees in Toronto, Canada.

**Methods:**

We conducted a retrospective chart review of 1042 refugee patients rostered at a specialized primary care clinic in Toronto from December 2011 to September 2016. Patients who completed recommended stool O&P analyses were included. Basic sociodemographic and clinical variables and results of stool O&P were examined.

**Results:**

419 patients (40.2%) had a stool O&P positive for any protozoan or helminth species. Sixty-nine patients (6.6%) had clinically significant parasite species (excluding *B hominis, D fragilis*, and *E dispar*, given their lower risk for causing symptoms/complications): 2.3% had clinically significant protozoans and 4.2% had helminths on stool analysis.

**Conclusion:**

Given the relatively low prevalence of clinically significant parasites identified, our findings do not support *universal* screening for enteric parasites with stool O&P among refugee claimants/asylum seekers. However, stool analysis should be considered in certain clinical situations, as part of a more tailored approach.

## Introduction

Globally, more than 82.4 million people were forcibly displaced in 2020—the highest numbers ever recorded [1]. Canada accepted 48,510 resettled refugees and asylum seekers in 2019 [2]. Refugees commonly arrive from regions with a higher burden of infectious diseases and may have endured social disruption, poor living conditions, and gaps in accessing health care [3–5]. Owing to these factors, refugees tend to be at higher risk for a range of infectious diseases, including intestinal parasitic infections.

While intestinal protozoal and helminth infections are relatively uncommon in the global north, they pose a significant burden to health worldwide [6]. The World Health Organization (WHO) estimates 1.5 billion people are infected with soil-transmitted helminth infections globally, with higher prevalence in tropical and subtropical regions [7]. Intestinal parasitic infections can result in a range of symptoms, including gastrointestinal symptoms and malnutrition, with potential impairment of growth and development among children, as well as more serious health complications [6, 7]. For example, the protozoan *Entamoeba histolytica* can cause severe amoebic colitis and liver abscesses [8, 9]. The intestinal helminth *Strongyloides stercoralis*, which can persist in human hosts for decades if untreated, can cause life-threatening disseminated disease in immunocompromised hosts [10, 11]. Chronic *Schistosomiasis mansoni* can result in liver fibrosis and other complications [12, 13]. *Ascaris lumbricoides* or *Trichuris trichiura* helminth infections in pregnant women can lead to maternal and neonatal complications [14–16].

Previous studies investigating the prevalence of intestinal parasites in fecal specimens among refugee populations arriving in higher income host countries have found rates ranging from 2 to 59% [17–23], with variation based on factors such as region of origin and age. Recommended strategies for enteric parasite screening and treatment also vary. Refugee health guidelines from the USA recommend presumptive pre-departure treatment for strongyloides and soil-transmitted helminth infections, as well as for schistosomiasis for those from sub-Saharan Africa, and either presumptive treatment or testing and treatment post-arrival, if needed based on clinical and individual factors [24]. Australian guidelines suggest serologic testing for strongyloides and for schistosomiasis (where appropriate, based on region of origin) and recommend pre-departure treatment of soil-transmitted helminths with albendazole [25]. For those who have not received pre-departure albendazole, the Australian guidelines recommend either presumptive treatment or screening with stool microscopy and directed treatment post-arrival. The Canadian “Evidence-based guidelines for immigrants and refugees” recommend screening for strongyloides and schistosomiasis with serologic tests for refugees from regions of high endemicity [26]. However, the guidelines do not address screening for other intestinal parasites with stool ova and parasite (O&P) testing. Additional data on the prevalence of intestinal parasites among newly arrived refugees can help guide clinical practice.

This study aims to investigate the prevalence and species of intestinal parasites identified in stool O&P among a large sample of newly arrived refugees in Toronto, Ontario.

## Material and methods

### Study design

We conducted a retrospective chart review of the electronic medical records (EMRs) of patients attending one of Canada’s largest specialized primary care clinics for refugees and refugee claimants (asylum seekers) in Toronto, Ontario. Any individual who enrolled at the clinic between November 2011 and September 2016, had one clinic visit, and completed requested stool O&P testing were included in the study; 1042 clinic patients met criteria. Among individuals rostered at the clinic during this time period, 1107 were excluded due to not completing stool O&P testing. The term “patients” refers to individuals who voluntarily registered with clinic for any reason for intake and thus became registered with the clinic for ongoing care; some individuals connected to the clinic to address a wide range of clinical symptoms (physical or mental health), while others did not report clinical concerns on intake but wanted to engage in preventative care or to be registered for primary care in case of future concerns.

Patients resided in the Greater Toronto Area (population 5.928 million in 2016, land area 5.906 km^2^) [27]. As context, in 2015, 20,046 refugees and 16,055 refugee claimants arrived in Canada and in 2016, 46,705 refugees and 23,860 claimants came to Canada [28, 29]. During the initial clinic visit, clinicians conducted a comprehensive medical history, including a structured discussion of key sociodemographic factors; completed a physical exam; and recommended standardized screening laboratory testing, including stool O&P testing, alongside other recommended tests such as complete blood count and hepatitis B serologies (drawing from the Canadian “Evidence-based guidelines for immigrants and refugees” [26]). Information was directly entered into patient EMRs by clinicians during the course of routine clinical care. A minority of clinic attendees were children of refugee parents, born in Canada, the USA, or countries in Western Europe during their parents ‘migration. However, stool O&P was not generally ordered for this patients unless they had spent time in countries with higher burden of intestinal parasites during their migratory journey. Stool O&P testing was otherwise ordered for all clinic attendees, regardless of the presence of symptoms. Data was subsequently extracted through automated searches and manual chart review. We collected data on the following variables: age, country of birth, country of origin, highest level of education completed, refugee category (refugee claimant, privately sponsored refugee [PSR], government-assisted refugee [GAR], and ‘other’ categories), length of time spent in Canada (based on date of arrival to Canada and date of first visit), pregnancy status of women of reproductive age, height, and weight. Laboratory test results of interest included: stool O&P results (including species of any parasites identified), hemoglobin, and eosinophil count, and were based on earliest available test results. Specific standardized screening questions regarding gastrointestinal symptoms were not a part of the intake assessment and thus the presence/absence of gastrointestinal or other relevant symptoms could not be included as a variable for analysis.

Patients were not subjected to, or deprived of, any additional testing or treatment. The study was approved by the Research Ethics Board of Women’s College Hospital.

### Definitions

The World Bank regional groupings according to the Atlas of Sustainable Development Goals 2018 were used to categorize patients’ countries of birth and countries of origin for sub-analyses [30]. We also introduced variables to indicate if country and region of birth differed from country and region of origin, respectively, which may reflect aspects of patients’ migratory pathways.

Reproductive age was defined as all women between 15 and 49 years of age in accordance with the WHO definition [31]. Body mass index (BMI), based on patients’ height and weight, was used to determine if patients were underweight. For patients aged 0 to 19 years, BMI values below the fifth percentile, based on WHO curves, were classified as underweight. For patients aged over 19 years, BMI values below 18.5 were considered underweight [32]. We used WHO anemia guidelines to define hemoglobin thresholds for the presence of anemia, which account for age, sex and pregnancy status for women of reproductive age; mild, moderate, and severe anemia were all classified as anemia in our study [33]. We defined eosinophilia as eosinophil count > 0.5 × 109 cells/liter, a commonly used threshold.

For stool O&P species, we distinguished between clinically significant protozoans and those considered to be commensal organisms or to have lower potential for pathogenicity. The latter category, termed here as “clinically less significant protozoans” include *Blastocystis hominis*, *Dientamoeba fragilis*, and *Entamoeba dispar*, which are often found in individuals without obvious clinical manifestations or complications, and therefore generally don’t require treatment in asymptomatic individuals.

### Lab testing

Patients were asked to submit three stool samples for analysis. Stool testing for ova and parasites was performed by Public Health Ontario Lab. Specimens are examined by microscopy by preparing a smear and concentrate after a centrifugation procedure utilizing the Formalin/Ethyl-acetate method [34]. Small numbers of organisms can be detected through the concentration method. A permanent stained smear, prepared from the concentrate, is used to identify trophozoites, occasionally cysts, and to confirm species. Iron Hematoxylin stain is used to identify and confirm non-coccidial intestinal protozoa. Positive microscopy results for *Entamoeba sp.* were additionally tested using enzyme-linked immunoassay (EIA) to differentiate between *Entamoeba histolytica* and *Entamoeba dispar *[35].

### Statistical analysis

Descriptive statistics including proportions, means, standard deviations (SD), medians, and interquartile ranges (IQR) were calculated to describe characteristics in our sample. Chi-square test was used to test categorical variables with the outcome of interest and the non-parametric Mann Whitney *U* (also known as Wilcoxon rank sum test) for testing metric and categorical variables. P-values less than 0.05 were considered as significant. Bivariate regression was used to test for associations between socio-demographic characteristics and the presence of parasites on stool microscopy. The quantification of effect sizes were reported as crude odds ratios (OR) with corresponding 95% confidence intervals (CI). Due to overall low numbers of positive test results we abstained to perform multivariate analyses. All statistical analyses were performed using SPSS (Version 25, IBM, Armonk NY). Figures and maps were constructed using Google Spreadsheet (Alphabet Inc., Mountain View, CA).

## Results

**Fig. 1:**
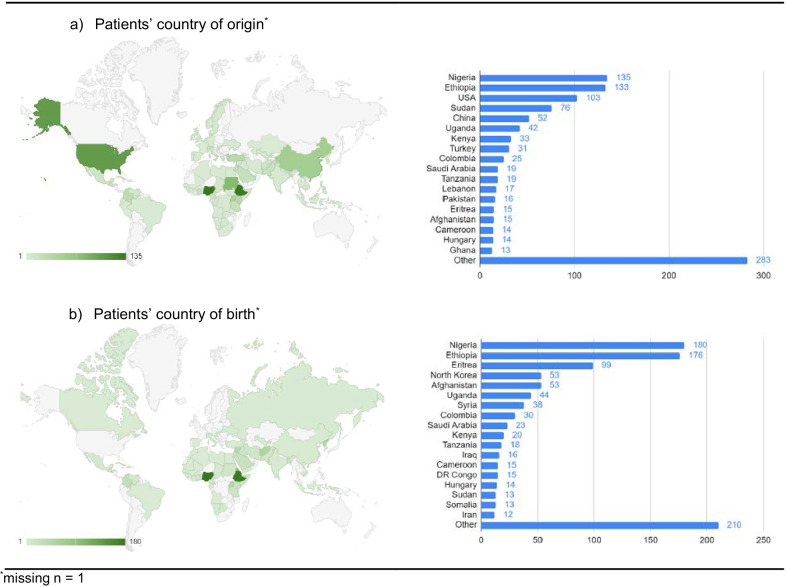
Countries of origin and birth of clinic patients

Our study included 1042 patients with a total of 3026 submitted stool samples. Approximately half of the patients (48.9%) were female and the median age at the initial clinical visit was 31 years (IQR 23–28; Table 1). Patients aged 30 to 39 years represented the largest age group in our sample. The median time between entry to Canada and the first visit at the clinic was 2 months (IQR 1–5 and maximum 51 months). Almost two-thirds (63.8%) of patients were from sub-Saharan Africa. Patients’ countries of birth and origin are shown in Fig. 1; top countries of birth included Nigeria, Ethiopia, and Eritrea. Patients had varied migration trajectories: for 58% of patients their country of origin was consistent with their country of birth and 79.8% patients’ region of birth was consistent with their region of origin. A sizeable number of patients (n = 103) first entered the United States before coming to Canada, for variable durations of time. The majority of patients were refugee claimants (92.8%), and almost two-third of patients had a high school degree (24.1%) or some post-secondary education (41.7%).

**Table 1:** Baseline characteristics of clinic patients and associated number of submitted stool samples

	Included patients n = 1042	
	n	%
Sex		
Male	532	51.1
Female	510	48.9
Age group (years)		
0–9	135	13.0
10–19	73	7.0
20–29	265	25.4
30–39	340	32.6
40–49	159	15.3
50–59	48	4.6
60–69	19	1.8
70+	3	0.3
Region of birth^b^		
East Asia & Pacific	69	6.6
Europe & Central Asia	37	3.6
Latin America & Caribbean	86	8.3
Middle East & North Africa	107	10.3
North America	2	0.2
South Asia	76	7.3
Sub-Saharan Africa	664	63.8
Region of origin^b^		
East Asia & Pacific	75	7.2
Europe & Central Asia	103	9.9
Latin America & Caribbean	84	8.1
Middle East & North Africa	82	7.9
North America	104	10.0
South Asia	37	3.6
Sub-Saharan Africa	556	53.4
Region of origin same as region of birth^b^	831	79.8
Country of origin same as country of birth^b^	604	58.0
Refugee category^b^		
Refugee claimant	966	92.8
Other^a^	75	7.2
Highest level of education^c^		
Child ≤ 18 years	199	19.5
None	12	1.2
Less than high school degree	139	13.6
Graduated high school	246	24.1
Some or completed university/post-secondary	426	41.7
Underweight BMI^d^	29	3.1
Anemia^e^	142	13.9
Eosinophil count^f^		
< 0.5	938	91.0
0.5–1.5	86	8.3
> 1.5	7	0.7
Pregnant^g^	85	22.5

^a^Other refugee categories include: government-assisted refugees (GARs), privately-sponsored refugees (PSRs), blended visa office referred refugees (BVORs); ^b^missing n = 1; ^c^missing n = 20; ^d^Underweight defined as BMI < 5%ile (WHO age and sex-adjusted curves) for children and adolescents up to age 19 and BMI < 18.5 for adults, missing n = 97; ^e^missing n = 22; ^f^missing n = 11; ^g^In reproductive aged females 15–49 years

A minority of patients (3.1%) were considered underweight. Anemia was present among 13.9% of patients and 9.0% had eosinophilia. Eighty-five patients in our study were pregnant at the time of first clinic visit, representing 22.5% of reproductive aged females (15–49 years). The vast majority (95%) of those who returned stool samples returned all 3 specimens. Slightly fewer specimens were returned among children under ten years old, females, those underweight, and patients who were born in the Middle Eastern region. However, in all these subgroups, an average > 2.8 samples were returned for lab analysis.

Intestinal parasites of any species were identified in samples submitted by 419 patients (40.2%) and 69 patients (6.6%) had clinically significant parasites on stool analysis. Only one patient had stool with multiple clinically relevant pathogens (*Strongyloides stercoralis* and *Taenia species*)*.*

### Protozoans

**Fig. 2:**
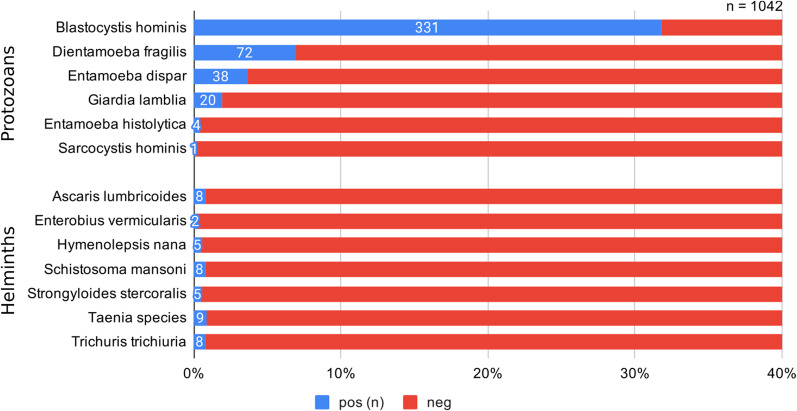
Frequency of parasite organisms identified in patients’ stool ova & parasite samples (n = 1042)

Four-hundred individuals (38.4%) had protozoans in their stool samples. The most common species were clinically less significant protozoans: *Blastocystis hominis* (n = 331), *Dientamoeba fragilis* (n = 72), and *Entamoeba dispar* (n = 38), which together represented 93.8% of all protozoan positive stool results (Fig. 2). Twenty-five patients (2.4%) had stool samples with clinically significant pathogens, including *Giardia lamblia* (n = 20), *Entamoeba histolytica* (n = 4)*,* and *Sarcocystis hominis* (n = 1). Higher rates of clinically significant protozoa were found among the 0 to 9 year old age group (5.2%, p = 0.023; Table 2). There was significant regional variation on bivariate analysis, with highest rates of clinically significant protozoa among patients from South Asia (9.2%, p < 0.001) and lowest rates among patients from sub-Saharan Africa (0.9%, p < 0.001). Increasing time in Canada was associated with lower rates of any protozoan positive stool samples (Mann–Whitney-*U* Z = − 4.544, p < 0.001), notably for *Giardia lamblia* (Mann–Whitney-*U* Z = − 2.004, p = 0.045) and *Blastocystis hominis* (Mann–Whitney-*U* Z = − 5.252, p < 0.001).

**Table 2:** Presence of protozoan and helminth parasites in patients’ stool samples, univariable and multivariable analyses (n = 1042)

	Protozoans				Helminths		
	Negative n = 642	Clinically less significant^a^ n = 375	Clinically significant pathogen^b^ n = 25	Clinically significant pathogens vs. negative and less significant protozoans	Negative n = 997	Positive n = 44	Positive vs. negative
	n (%)	n (%)	n (%)	OR (95% CI)	n (%)	n (%)	OR (95% CI)
Sex							
Male	305 (57.3)	216 (40.6)	11 (2.1)	1	502 (94.4)	30 (5.6)	1
Female	337 (66.1)	159 (31.1)	14 (2.8)	1.01 (0.99–1.03)	496 (97.3)	14 (2.7)	0.97 (0.95–1.00)
Age							
0–9	98 (72.6)	30 (22.2)	7 (5.2)	1.05 (1.02–1.08)	131 (97.0)	4 (3.0)	0.99 (0.95–1.03)
10–19	55 (75.0)	15 (20.5)	3 (4.1)	1.04 (1.00–1.08)	69 (94.5)	4 (5.5)	1.02 (0.97–1.07)
20–29	150 (56.6)	107 (40.4)	8 (3.0)	1.02 (1.00–1.05)	252 (95.1)	13 (4.9)	1.01 (0.98–1.04)
30–39	199 (58.5)	139 (40.9)	2 (0.6)	1	327 (96.2)	13 (3.8)	1
40–49	96 (60.4)	61 (38.4)	2 (1.3)	1.01 (0.98–1.04)	150 (94.3)	9 (5.7)	1.02 (0.98–1.06)
50–59	29 (60.4)	17 (35.4)	2 (4.2)	1.04 (0.99–1.09)	47 (97.9)	1 (2.1)	0.98 (0.92–1.04)
60–69	14 (73.3)	4 (21.1)	1 (5.3)	1.05 (0.98–1.12)	19 (100)	0 (0.0)	0.96 (0.88–1.06)
70+	1 (33.3)	2 (66.7)	0 (0.0)	0.99 (0.84–1.18)	3 (100)	0 (0.0)	0.96 (0.77–1.21)
Region of birth^d^							
East Asia & Pacific	60 (87.0)	8 (11.6)	1 (1.4)	1.01 (0.97–1.04)	64 (92.8)	5 (7.2)	1.02 (0.97–1.07)
Europe & Central Asia	31 (83.8)	5 (13.5)	1 (2.7)	1.02 (0.97–1.07)	37 (100)	0 (0.0)	0.95 (0.89–1.02)
Latin America & Caribbean	49 (57.0)	33 (38.4)	4 (4.7)	1.04 (1.00–1.07)	84 (97.7)	2 (2.3)	0.97 (0.93–1.02)
Middle East & North Africa	63 (58.9)	38 (35.5)	6 (5.6)	1.05 (1.02–1.08)	107 (100)	0 (0.0)	0.95 (0.91–0.99)
North America	2 (100)	0 (0.0)	0 (0.0)	0.99 (0.80–1.22)	2 (100)	0 (0.0)	0.95 (0.72–1.26)
South Asia	38 (50.0)	31 (40.8)	7 (9.2)	1.09 (1.05–1.13)	73 (96.1)	3 (3.9)	0.99 (0.94–1.04)
Sub-Saharan Africa	398 (59.9)	260 (39.2)	6 (0.9)	1	631 (94.9)	34 (5.1)	1
Region of origin^d^							
East Asia & Pacific	65 (86.7)	9 (12.0)	1 (1.3)	1.00 (0.97–1.04)	70 (93.3)	5 (6.7)	1.01 (0.96–1.06)
Europe & Central Asia	74 (71.8)	28 (27.2)	1 (1.0)	1.00 (0.97–1.03)	103 (100)	0 (0.0)	0.95 (0.91–0.99)
Latin America & Caribbean	47 (56.0)	34 (40.5)	3 (3.6)	1.03 (0.99–1.06)	82 (97.6)	2 (2.4)	0.97 (0.93–1.02)
Middle East & North Africa	43 (52.4)	33 (40.2)	6 (7.3)	1.06 (1.03–1.10)	81 (98.8)	1 (1.2)	0.96 (0.92–1.00)
North America	64 (61.5)	36 (34.6)	4 (3.8)	1.03 (1.00–1.06)	99 (95.2)	5 (4.8)	0.99 (0.95–1.04)
South Asia	17 (45.9)	16 (43.2)	4 (10.8)	1.10 (1.05–1.16)	36 (97.3)	1 (2.7)	0.97 (0.91–1.04)
Sub-Saharan Africa	332 (59.7)	218 (39.2)	6 (1.1)	1	526 (94.6)	30 (5.4)	1
Refugee category							
Refugee claimant	599 (62.0)	347 (35.9)	20 (2.1)	1	922 (95.4)	44 (4.6)	1
Other^c^	43 (57.3)	27 (36.0)	5 (6.7)	1.05 (1.01–1.09)	75 (100)	0 (0.0)	0.96 (0.91–1.00)
Region of origin same as region of birth^d^	510 (61.4)	300 (36.1)	21 (2.5)	1.01 (0.98–1.03)	793 (95.4)	38 (4.6)	1.02 (0.99–1.05)
Country of origin same as country of birth^d^	351 (58.1)	238 (39.4)	15 (2.5)	1.00 (0.98–1.02)	575 (95.2)	29 (4.8)	1.01 (0.99–1.04)
Level of education^e^							
Children ≤ 18 years	148 (74.4)	41 (20.6)	10 (5.0)	1.03 (1.01–1.06)	191 (96.0)	8 (4.0)	0.99 (0.96–1.03)
None	7 (58.3)	4 (33.3)	1 (8.3)	1.07 (0.98–1.17)	12 (100)	0 (0.0)	0.95 (0.85–1.07)
Less than high school	86 (61.8)	49 (35.3)	4 (2.9)	1.01 (0.98–1.04)	136 (97.8)	3 (2.2)	0.97 (0.94–1.01)
Graduated high school	167 (67.9)	76 (30.9)	3 (1.2)	1.00 (0.97–1.02)	234 (95.1)	12 (4.9)	1.00 (0.97–1.03)
Some or completed university/post-secondary	222 (52.1)	197 (46.2)	7 (1.6)	1	405 (95.1)	21 (4.9)	1
Underweight BMI^f^	19 (65.5)	8 (27.6)	2 (6.9)	1.05 (0.99–1.11)	27 (93.1)	2 (6.9)	1.03 (0.95–1.11)
Anemia^g^	92 (64.8)	49 (34.5)	1 (0.7)	0.98 (0.96–1.01)	138 (97.2)	4 (2.8)	0.98 (0.95–1.02)
Eosinophil count^h^							
< 0.5	583 (62.2)	335 (35.7)	20 (2.1)	1	908 (96.8)	30 (3.2)	1
0.5–1.5	52 (60.5)	30 (34.9)	4 (4.7)	1.03 (0.99–1.06)	74 (86.0)	12 (14.0)	1.11 (1.07–1.16)
> 1.5	1 (14.3)	6 (85.7)	0 (0.0)	0.98 (0.87–1.1)	5 (71.4)	2 (28.6)	1.29 (1.11–1.49)
Pregnant^i^	58 (68.2)	25 (29.4)	2 (2.4)	1.01 (0.99–1.04)	81 (95.3)	4 (4.7)	1.02 (0.98–1.06)

^a^“Clinically less significant protozoans” includes *Blastocystis hominis*, *Dientamoeba fragilis*, and *Entamoeba dispar*; ^b^“Clinically significant pathogen” here includes *Giardia lamblia*, *Entamoeba histolytica*, and *Sarcocystis hominis*; ^c^Other refugee categories include: GARs, PSRs, BVORs; ^d^missing n = 1; ^e^missing n = 20; ^f^Underweight defined as BMI < 5%ile (WHO age and sex-adjusted curves) for children and adolescents up to age 19 and BMI < 18.5 for adults, missing n = 97; ^g^missing n = 22; ^h^missing n = 11, 10^9^ cells/liter; ^i^In reproductive aged females 15–49 years

**Fig. 3:**
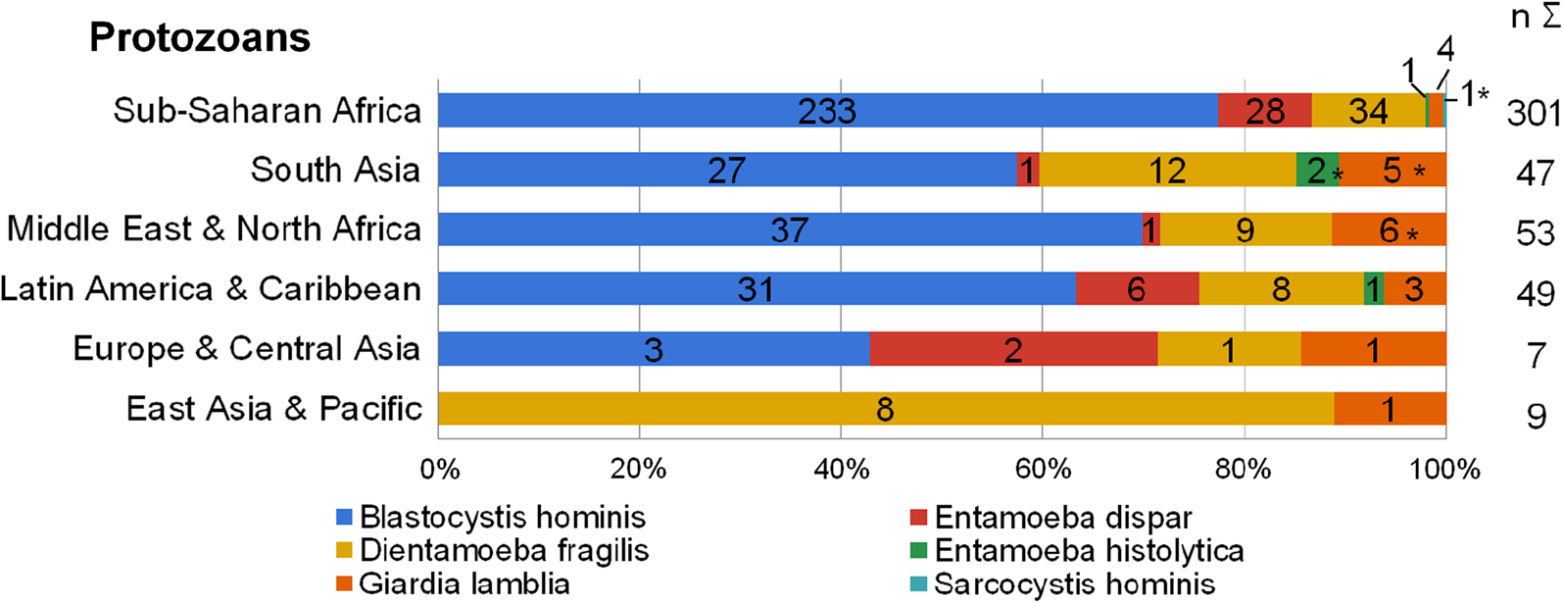
Parasite species detected in patients’ stool by patients’ region of birth

*Entamoeba histolytica* was significantly more common in patients born in South Asia (2.6% vs. 0.2% in non-South-Asians, p = 0.001; Fig. 3). *Giardia lamblia* was more commonly found in patients from the Middle East (5.6% vs. 1.5% in non-Middle-East, p = 0.003) and South Asia (6.6% vs. 1.6% in non-South-Asians, p = 0.002) and less often in patients from Africa (0.6% vs. 4.2% in non-African, p < 0.001). *Giardia lamblia* was more common among children aged 0 to 9 years (5.2% vs. 1.4% in other age groups, p = 0.003).

### Helminths

Forty-four (4.2%) patients had helminth species identified in their stool samples. Significantly higher rates were identified among males (5.6% vs. 2.7%, p = 0.020; Table 2). Most common species were *Taenia spp.* (n = 9), followed by *Trichuris trichiura*, *Schistosoma mansoni*, and *Ascaris lumbricoides* (each n = 8).

In the bivariate analyses, patients’ region of birth was not significantly correlated with the presence of helminths, except that no helminth species were found among patients from the Middle East & North Africa region (p = 0.022). Patients originating from Europe & Central Asia (region of origin) were less likely to have helminths (OR 0.95, 95% CI 0.91–0.99). Eosinophilia was associated with a higher likelihood of helminth infestation, with even higher odds ratio among patients with higher eosinophilia counts > 1.5 × 10^9^ cells/liter (OR 1.25, 95% CI 1.11–1.49).

Specific helminth species varied by certain subgroups. *Schistosoma mansoni* and *Taenia spp.* were more prevalent in patients from sub-Saharan Africa (1.2% vs. 0.0% in non-sub-Saharan, p = 0.032 and 1.4% vs. 0.0% in non-sub-Saharan, p = 0.023, respectively), whereas rates of *Ascaris lumbricoides* were higher among patients from South Asia (4.0% vs 0.5% in non-South Asians, p = 0.001). *Trichuris trichiuria* was more common in patients born in the East Asian and Pacific region (7.3% vs. 0.3% in non-East Asians, p < 0.001). *Enterobius vermicularis* was more often found in 10 to 19-year-olds (1.4% vs 0.1% in other age groups, p = 0.017).

## Discussion

In our study population of newly arrived refugees (mostly claimants), 40.2% patients had a stool sample positive for any protozoan or helminth species. Only 6.6% of all patients had clinically significant parasite species (excluding *B hominis, D fragilis*, and *E dispar*, given their lower risk for causing symptoms/complications): 2.3% had clinically significant protozoans and 4.2% had helminths on stool analysis. The prevalence of intestinal parasites found in our study is much lower than reported in many patients’ countries of birth and origin [6, 36, 37]. Various factors may contribute to these differences, including the natural history of many intestinal parasites clearing with time without intervention.

Our findings show a higher prevalence of positive stool samples of any parasite species than recently reported by DeVetten et al. [20] in a similar clinical context in Canada, where they found an overall intestinal parasite prevalence of 29.7% in refugee newcomer patients at a primary care clinic in Calgary. However, when *B hominis* and *D fragilis* were excluded, the prevalence of parasites was only 16.3% [20]. Our sample included mostly refugee claimants (92.7%) whereas the majority of patients in the Calgary study were government-assisted or privately sponsored refugees (88%). Migratory journeys, prior living conditions, countries of origin, and health conditions and needs often differ among different refugee categories, which may contribute to the differences in observed stool-based prevalence of parasites [38, 39]. A recent study of newly arrived asylum seekers in Italy found prevalence rates of intestinal parasites of 20.6%, of which the majority (83%) were protozoa, including *B hominis* [22]. In a sample of asylum seekers in Washington, D.C. the prevalence of pathogenic parasites was much lower at 4%, excluding *B hominis and E dispar* [18]. Some earlier studies with data from the 1990s reported higher rates of positive stool samples among refugee newcomers—up to 58.6% in some regions [17, 40]. Decreasing rates of parasitic infections, and helminth species in particular, may reflect progress from the WHO’s mass deworming campaigns and improvements in water and sanitation in recent decades [22].

While 38.4% of patients had protozoans detected in their stool samples, clinically significant protozoal pathogens—*Entamoeba histolytica*, *Giardia lamblia,* and *Sarcocystis hominis*—were rare (2.4%). Giardia lamblia was the most common pathogenic protozoa, similar to findings in other studies [20, 21, 40–42]. We found higher prevalence rates of clinically significant protozoal pathogens in children and youth aged 0 to 9 years and 10 to 19 years, consistent with other studies that have found higher rates of protozoa among children [21, 40, 43]. The prevalence of clinically significant protozoans was also higher in patients who were born in South Asia, Latin America & the Caribbean, and Middle East & North Africa. Although protozoan infections are endemic in many parts of sub-Sarahan Africa [36, 44], we found a relatively low prevalence (0.9%) of clinically significant protozoans in patients from this region.

Helminth species were identified in 4.2% of all screened individuals. Patients born in the East Asian & Pacific region showed higher helminth prevalence rates than patients born in sub-Saharan African (7.2% vs 5.1%). *Taenia spp.* followed by *Trichuris, Schistosoma, and Ascaris* were the most commonly identified helminths. Other studies have found a variety of helminth species on stool analysis, with a tendency towards a predominance of *Trichuria* and hookworm in several studies [21, 45, 46]. In our study, *Schistosoma mansoni* and *Taenia spp.* were more often found among patients from sub-Saharan Africa, while *Ascaris lumbricoides* was more prevalent among patients from South Asia.

Eosinophilia correlated significantly with the presence of helminth species on stool analysis and, as would be predicted, was not significantly associated with the presence of protozoan species. Other studies have similarly identified associations of eosinophilia with helminth infections among refugee patients [41, 42]. Eosinophilia among refugees from endemic countries should raise clinical suspicion for enteric parasite infection and for helminths (particularly *S. stercoralis*). Underweight BMI and anemia were not associated with the presence of clinically significant protozoan species or helminths, although assessment for enteric parasites should be considered among refugee patients with these clinical markers and without another cause. Other studies have found poor correlation of gastrointestinal symptoms with intestinal parasite infections [40, 47], but research is limited in this area and further investigation is needed.

The detected rates of intestinal schistosomiasis and strongyloides infections are likely substantial underestimates, as the sensitivity of stool O&P is relatively low for detecting these species [12, 13, 48]. Serological assays have higher sensitivity and are the preferred test for detecting these species (although they may overestimate prevalence). A recent meta-analysis of strongyloides and schistosomiasis prevalence among migrants born in endemic areas found a pooled strongyloides seroprevalence of 12.2% (95% CI 9.0–15.9%) and stool-based prevalence of 1.8% (95% CI 1.2–2.6%) and schistosomiasis seroprevalence of 18.4% (95% CI 13.1–24.5%) and stool-based prevalence of 0.9% (95% CI 0.2–1.9%). Early identification and treatment of strongyloides and schistomiasis is important because, unlike most parasites, these species can persist in human hosts for decades and can lead to significant morbidity and mortality if untreated [12]. Treatment for both infections are short and well-tolerated and are associated with moderate to high cure rates: 83–100% with ivermectin for strongyloides and 52–92% with praziquantel for *S. mansoni* [12].

Additionally, lower rates of detection of *Enterobius vermicularis* in our study are likely because this organism is detected using an adhesive strip test and not by analyzing stool samples. The prevalence of *Enterobius vermicularis* may be higher than reported, especially among children in our sample, but were not detected by stool analysis.

Of note, conventional and real-time polymerase chain reaction (PCR) techniques offer a sensitive and specific alternative to microscopic examination for intestinal helminth and protozoan detection in stool samples [49]. Ongoing development of these molecular techniques may improve detection of intestinal parasites.

## Strengths and limitations

This study involves a large cohort of patients enrolled over a five year period from a broad geographic range. The majority of patients provided three stool samples for analysis providing a large study sample. Additionally, the Public Health Ontario laboratory was able to discriminate between the pathogenic *Entamoeba histolytica* from the less pathogenic *Entamoeba dispar* by using ELISA based test methods. This adds significantly to existing literature, as this differentiation is often not available [17, 19, 20, 43, 50].

Our study has several limitations. Study participants included patients who voluntarily sought care at a primary care clinic and completed recommended medical screening exams, which may introduce a selection bias. Additionally, this sample of patients, while large, reflects demographics and geographic distribution of primarily refugee claimants to a single centre during a particular period of migration to Canada, whereas migratory patterns are dynamic. Results cannot necessarily be generalized to the broader refugee newcomer population. Additional sociodemographic characteristics, such as prior living conditions (e.g. rural vs urban), and additional clinical variables, such as the presence of symptoms at the time of stool testing, could be useful for further analysis but were not available. Although we had a large number of participants, conclusions drawn from the sub-analysis by sociodemographic factors and by species are limited by the low number of clinically significant parasites that were detected.

## Conclusion

Our study showed relatively high prevalence rates for any intestinal parasite (40.2%) but low prevalence of clinically significant pathogens (6.6%). The observed prevalence of enteric parasites is much lower than the rates reported in patients’ countries of birth and countries of origin, likely owing to the natural history of self-clearance of most parasites. Given low prevalence rates of clinically significant parasites, our findings do not support *universal* screening for enteric parasites with stool O&P among refugee claimants. However, stool analysis should be considered in the presence of eosinophilia; in certain clinical situations, including gastrointestinal symptoms, weight loss or growth concerns, and anemia; and individual circumstances, such as recent exposure to poor sanitation. Screening for strongyloides and schistosomiasis (where appropriate based on region of origin) through serologic testing remain important measures for detecting and managing parasitic infections with higher risk of severe morbidity and mortality. Ongoing studies investigating enteric parasite screening and diagnosis among refugees would help to further refine clinical care and guidelines.

## Data Availability

The dataset is not publicly available as this is not approved by the responsible research ethics board. However, the datasets generated and analysed are available from the authors on reasonable request within a data sharing agreement and additional ethic board approval.
